# Supplementary figures and images for: Visualizing synaptic dopamine efflux with a 2D composite nanofilm
Source: eLife. 2022 Jul 4;11:e78773. doi: 10.7554/eLife.78773 (PMC9363124; doi:10.7554/eLife.78773)

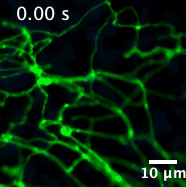

Supplement: Supplementary file 1 [file elife-78773-fig2-video1.gif]

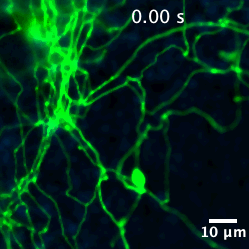

Supplement: Supplementary file 2 [file elife-78773-fig4-video1.gif]

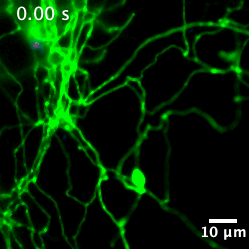

Supplement: Supplementary file 3 [file elife-78773-fig4-video2.gif]

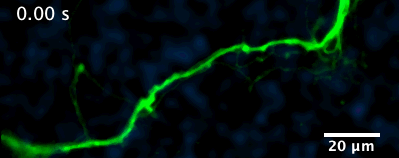

Supplement: Supplementary file 4 [file elife-78773-fig5-video1.gif]

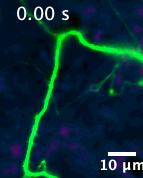

Supplement: Supplementary file 5 [file elife-78773-fig5-video2.gif]

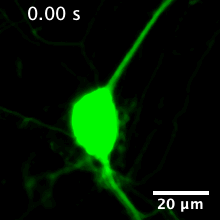

Supplement: Supplementary file 6 [file elife-78773-fig5-video3.gif]
